# Laser Versus Rotational Atherectomy in Coronary Artery Disease: A Systematic Review and Meta-Analysis of Procedural Success and Safety

**DOI:** 10.7759/cureus.84832

**Published:** 2025-05-26

**Authors:** Moiuz Chaudhri, Mohamed Ellebedy, Ahmed D Al Mahrizi, Muhammad F Ali, Han J Liu, Matthew Boyle, Barira Haroon, Ogechukwu Obi, Fredrick Acquah, Louise Sakowski, Mohammed Nagaria, John Rickards, Ayesha Samad, John Girges, Neil Patel, Shrujal A Parikh, Christian Kaunzinger, Muhammad R Raza, Aditya Mehra

**Affiliations:** 1 Cardiology, Hackensack Meridian Ocean University Medical Center, Brick Township, USA; 2 Internal Medicine, Faculty of Medicine, Sohag University, Sohag, EGY; 3 Internal Medicine, Faculty of Medicine and Surgery, University of Malta, Msida, MLT; 4 Internal Medicine, Department of Medicine, Jinnah Postgraduate Medical Center, Karachi, PAK; 5 Cardiology, Rowan-Virtua School of Osteopathic Medicine, Stratford, USA; 6 Internal Medicine, Rowan-Virtua School of Osteopathic Medicine, Stratford, USA; 7 Internal Medicine, New York Institute of Technology College of Osteopathic Medicine, Old Westbury, USA; 8 Cardiology, Piedmont Macon Medical Center, Macon, USA; 9 Cardiology, Mercer University School of Medicine, Macon, Georgia, USA; 10 Internal Medicine, Hackensack Meridian Ocean University Medical Center, Brick Township, USA; 11 Internal Medicine, Hackensack Meridian Ocean Medical Center, Brick Township, USA

**Keywords:** coronary calcification, laser atherectomy, plaque debulking, procedural outcomes, rotational atherectomy, stent optimization

## Abstract

The evolution of percutaneous coronary intervention (PCI) has improved the management of complex coronary lesions, particularly in heart failure patients. Laser atherectomy (LA) and rotational atherectomy (RA) are used to treat in-stent restenosis and calcified stenosis. Both techniques share similar indications and risks, but direct comparisons of their efficacy and safety are limited. This review examines procedural success, complication rates, and clinical outcomes of RA and LA. PubMed, Embase, and the Cochrane Library were searched to retrieve studies between 2015 and 2025. Primary outcomes included procedural success, major adverse cardiovascular events (MACE), and complications, including dissection and perforation. Random-effects models were used for analysis, with subgroup analyses based on lesion type and complexity. Fourteen studies were included in our meta-analysis (LA: 6 studies; RA: 8 studies). LA showed a procedural success rate of 96.3%, higher than RA (93.3%). The increase in lumen diameter after the procedure was statistically significantly higher in LA (mean difference: 6.71 mm²; 95% CI: (6.64-6.79); p < 0.001) as compared to RA (mean difference: -27.90 mm²; 95% CI: (-27.95,-27.85); p < 0.001). Subgroup analysis revealed that RA worked better on severely calcified lesions that required stentablation. Complication rates were similar between the two techniques (1.2% for LA vs. 1.5% for RA; p = 0.21). LA provides better procedural success and lumen gain in heart failure patients with complex coronary lesions. However, RA remains superior for stentablation in non-dilatable, calcified lesions. Both techniques have similar safety profiles, suggesting the need for individualized treatment based on patient and lesion characteristics.

## Introduction and background

The presence of heavily calcified lesions, in-stent restenosis (ISR), and underexpressed stents is commonly found in patients with a diagnosis of complex coronary artery disease (CAD). There is an ongoing challenge in the use of percutaneous coronary intervention (PCI) in this scenario. There is insufficient information regarding the preparation of lesions in these anatomically inaccessible sites, which makes the use of traditional balloon angioplasty and stent deployment less effective. This leads to failure of the stent due to failure of the target lesion, poor balloon expansion, and thrombosis formation [1,2]. To overcome these obstacles, several technologies involving atheroablative techniques, such as excimer laser coronary atherectomy (ELCA) and rotational atherectomy (RA), have served as supplementary approaches. ELCA uses pulsed ultraviolet light to lower neointimal hyperplasia and thrombotic material while maintaining the integrity of nearby tissues. Several observational studies have established its effectiveness and efficacy in patients with ISR, saphenous vein graft lesions, and calcified lesions, displaying favorable midterm outcomes [3-8].

Results from recent studies have suggested good technical success and procedure-related outcomes when utilizing contrast mix injection or optical coherence tomography-guided implantation [3,7]. However, rotational atherectomy uses an accelerated rotating rough edge to modify plaque at the interface, which helps improve the dilation of noncompliant and heavily calcified lesions for optimal expansion of the stent. Notwithstanding that RA has been employed in interventional cardiology for a longer duration, its application in ISR has produced inconsistent outcomes, with some studies, such as the ARTIST study, indicating no significant advantages over conventional angioplasties [1]. Recently, novel applications like stentablation, utilizing RA to modify underexpanded or inadequately expanded stents, have demonstrated positive outcomes with a high procedural success rate [8,9]. Additionally, the implantation of drug-eluting stents with RA for calcified lesions appears to lower repeat revascularization rates compared to bare-metal stents [10].

Despite the growing clinical experience with ELCA and RA in complex coronary contexts, the comparative efficacy and safety of ECLA and RA, particularly concerning the dual issues of ISR and stent under expansion, is yet to be clarified. As a result, we conducted a meta-analysis of state-of-the-art studies evaluating the efficacy and safety of ELCA and RA in complex coronary interventions, focusing on their effects on clinical outcomes such as target lesion revascularization, major adverse cardiovascular events (MACE), and overall mortality.

## Review

Protocol registration

The current systematic review and meta-analysis were performed in compliance with the Preferred Reporting Items for Systematic Reviews and Meta-Analyses (PRISMA) 2020 guideline [11]. The protocol for the study is registered on PROSPERO (CRD42025634990). The study aimed to compare MACE and complications such as coronary dissection and perforation, and restenosis rate requiring revascularization. We also compared procedural success, defined as less than 50% residual stenosis.

Data sources and study selection

A systematic search of PubMed, Embase, and the Cochrane Library was conducted to retrieve studies published between 2015 and 2025. Our search was done on January 7, 2025, by MC, MB, BH, and JL. The search strategy used a combination of Medical Subject Headings (MeSH) terms and free-text keywords for laser atherectomy, rotational atherectomy, coronary calcification, and clinical endpoints. Gray literature and references in relevant articles were also reviewed. Eligible studies were defined as clinical trials, cohort studies, and case-control studies that recruited adult patients (≥ 18 years) receiving PCI of either LA or RA. Investigators had to report at least one primary outcome.

Data extraction and quality assessment

Data extraction was performed independently by three authors according to a pre-specified data extraction guidelines, which included procedures with success with residual stenosis less than 50%, studies comparing adults undergoing percutaneous coronary intervention with either laser or rotational atherectomy, and reporting at least one MACE event. The variables extracted were study design, patient demographics, lesion characteristics, details of intervention, and clinical outcomes. Any discrepancies in the extracted data were resolved by discussion.

Risk of bias was assessed by two authors independently using the Cochrane risk-of-bias tool for randomized trials (RoB2) for the included RCTs [12], and Risk Of Bias In Non-randomized Studies-of Interventions (ROBINS-I) for non-randomized studies [13].

Statistical analysis

A meta-analysis was conducted using the random-effects model to allow for clinical and methodological diversity. Results were presented as risk ratios (RRs) for dichotomous outcomes and as mean differences (MDs) for continuous outcomes, along with 95% confidence intervals (CIs). Heterogeneity was measured with the I² statistic, where 25%, 50%, and 75% were considered to correspond to low, moderate, and high levels of heterogeneity, respectively. Subgroup analysis was conducted depending on the lesion characteristics (severe calcification and use of stentablation). Sensitivity analyses were performed to investigate the stability of the results, especially because of the heterogeneity observed, which was so pronounced.

The I² statistic was used to assess heterogeneity among studies, and the examining forest plot was also conducted. To explore possible heterogeneity and find studies that made outsize contributions to the inconsistency, we conducted a Baujat plot analysis. Baujat plots present graphically the contribution of each study to the overall heterogeneity (as assessed by the Q test) in relation to its influence on the overall summary OR. Studies in the upper right quarter of the Baujat plot were defined as potential outliers or large contributors of heterogeneity and underwent additional sensitivity analyses. Sensitivity analysis was further performed using a sequential exclusion of studies to explore the stability of the primary results and estimate the possible risk of bias of heterogeneity.

Results

Study Selection

**Figure 1:**
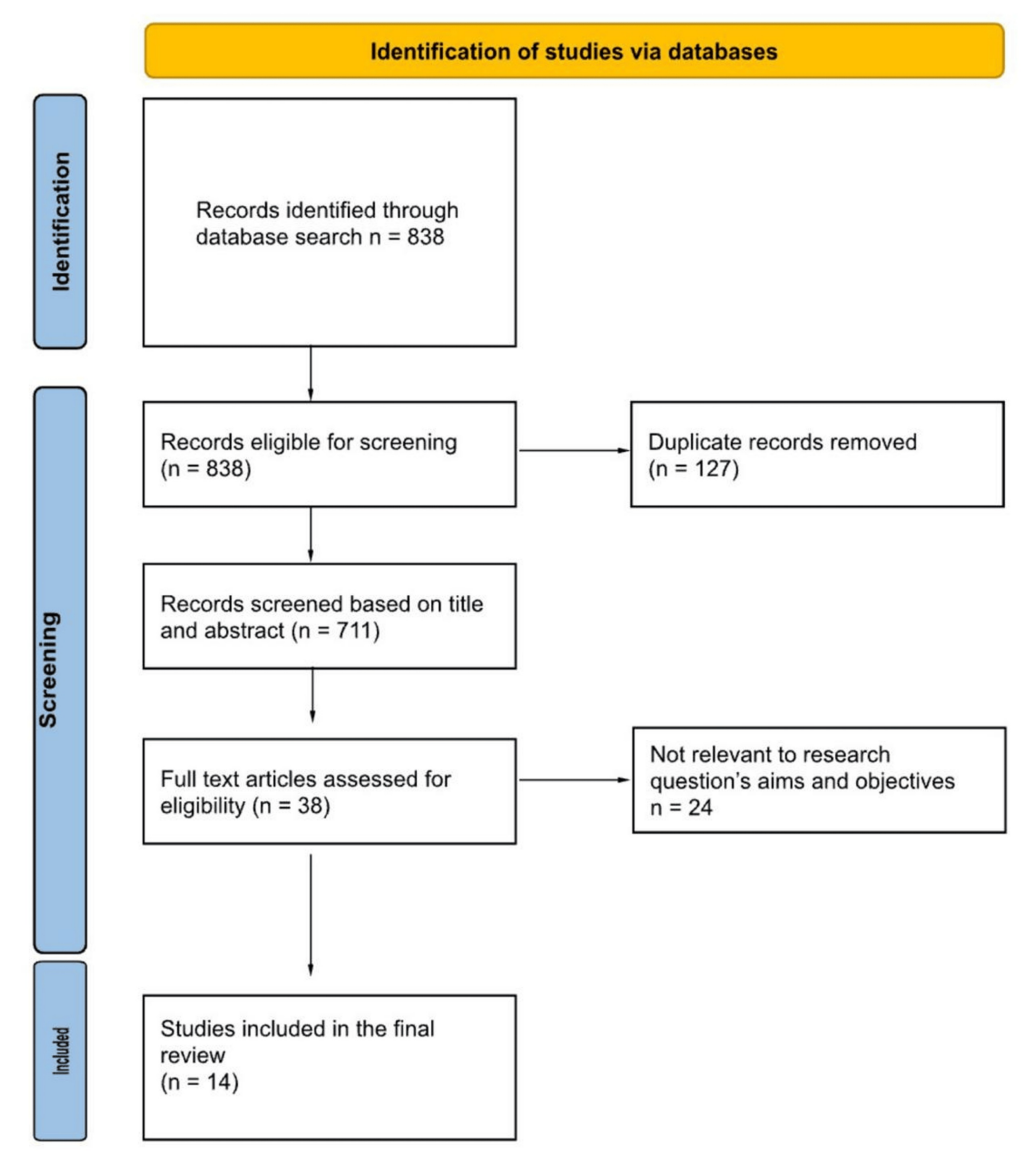
The PRISMA flow diagram of the study selection PRISMA: Preferred Reporting Items for Systematic Reviews and Meta-Analyses

A total of 838 studies were included in our research. After removing the 127 duplicates, 711 articles were reviewed by title and abstract, and 38 were included for full-text review. Twenty-four studies were eliminated, as they did not correspond to the research questions and goals. Two independent reviewers selected studies, with consensus or adjudication by a third reviewer if necessary (Figure 1).

Study Characteristics

Fourteen studies, with a total sample size of 2,822 patients, were included in the meta-analysis [1-3,6,7,10,14-21]. The studies included in this meta-analysis compared procedural results between LA and RA. The enrolled studies were different in nature and sample size, which were predominantly of patients with a calcified coronary artery and who needed stent intervention. Characteristics of the included studies are summarized in Table 1.

**Table 1:** Summary of the included studies

Author	Year	Country	Study Design	Sample Size (n)	Mean Age (years)	Male (%)	Hypertension (%)	Diabetes Mellitus (%)	Hyperlipidemia (%)	Prior Coronary Artery Bypass Grafting (%)	Chronic Renal Insufficiency (%)	Peripheral Vascular Disease (%)	Smoking Status	Population/Condition	Intervention	Comparator	Primary Outcome(s)	Follow-up Duration
Ali et al. [20]	2024	Egypt	Retrospective, Multicenter	49	61 ± 5.5	57.10%	63.30%	67.30%	44.90%	Not reported	Not reported	Not reported	61.20%	Peripheral artery in-stent restenosis	Mechanical rotational atherectomy + DCB	Drug-coated balloon alone	Technical success, patency, CD-TLR, mortality at 6 months	6 months
Ai et al. [19]	2018	China	Retrospective, Single-center	127	65.5	76.40%	66.70%	62.70%	34.70%	Not reported	4.0% (RA+CB) / 5.8% (RA only)	Not reported	61.3% (RA+CB) / 36.5% (RA only)	Calcified coronary lesions	Rotational atherectomy + Cutting balloon	Rotational atherectomy + Plain balloon	Acute lumen gain, final lumen diameter, ISR >1 year	>1 year
Dietz et al. [21]	2002	Europe (Multicenter)	Randomized Controlled Trial	298	60 (PTCA) / 62 (PTCR)	82% (PTCA) / 79% (PTCR)	Not reported	25% (PTCA) / 26% (PTCR)	Not reported	Not reported	Not reported	Not reported	Not reported	Diffuse in-stent restenosis	Rotational atherectomy	Balloon angioplasty	Minimum lumen diameter at 6 months	6 months
Reifart et al. [21]	1997	Germany	Randomized Controlled Trial	685	Not clearly reported	Not clearly reported	Not clearly reported	Not clearly reported	Not clearly reported	Not clearly reported	Not clearly reported	Not clearly reported	Not clearly reported	Complex coronary lesions	Rotational atherectomy	Balloon angioplasty	Procedural success, revascularization rates	6 months
Yasumura et al. [17]	2022	USA	Retrospective, Single-center	26	68.9 ± 7.7	61.50%	100%	61.50%	92.30%	34.60%	26.90%	11.50%	30.8% (current or former)	Undilatable in-stent restenosis	Rotational atherectomy	None (single-arm study)	Procedural success, MACE	1 year
Édes et al. [14]	2016	Hungary	Prospective Registry	12	70.8 ± 6.9	75%	100%	75%	91.70%	25%	Not reported	Not reported	Not reported	Undilatable stent lesions	Stentablation by rotational atherectomy	None (single-arm study)	Procedural success, MACE, mortality	6 months
Whiteside et al. [8]	2019	USA	Retrospective, Single-center	20	66.6 ± 9.4	75%	85%	60%	70%	35%	Not directly reported (eGFR given: 76.5 ± 24.1)	Not reported	Not reported	Undilatable coronary stents	Stentablation by rotational atherectomy	None (single-arm study)	Procedural success, MACE at 12 months	12 months
Pereira et al. [7]	2021	USA	Prospective, Single-operator	13	65 ± 11.2	83%	100%	50%	100%	16.70%	Not reported	Not reported	50% of current smokers	In-stent restenosis treated with ELCA + BVS	Excimer laser coronary atherectomy + BVS	None (single-arm study)	Technical success, MACE at 6 months	6 months
Köster et al. 15]	2000	Germany	Prospective, Single-center	96	60 ± 10	84%	66%	28%	75%	Not reported	Not reported	Not reported	55% (current or recent)	Coronary in-stent restenosis	Excimer laser coronary angioplasty	None (single-arm study)	Clinical and angiographic restenosis rates at 6 months	6 months
Ayoub et al. [2]	2023	Germany, Switzerland	Retrospective, Registry-based	193	70.3 ± 9.0 (RA group)	80.8% (RA group)	92.50%	43.40%	92.3% (dyslipidemia)	33.50%	eGFR lower in the RA group (66.2) but CKD not separately reported	12.3% (prior CVD)	10.99% current smokers	Chronic total occlusion	Rotational atherectomy	Patients undergoing CTO PCI without rotational atherectomy	Procedural success, MACCE at 1 year	1 year
Tamekiyo et al. [10]	2009	Japan	Observational, single-center	704	70.3 ± 10.5	57.80%	74.40%	49.3% (diabetes), 12.1% insulin-dependent	Not clearly separated; general high prevalence of dyslipidemia	8.50%	35.9% on dialysis (severe CKD)	Not reported	Not reported	Calcified coronary lesions treated with ROTA and sirolimus-eluting stents	Rotational atherectomy + sirolimus-eluting stent	Historical bare metal stent cohort with and without ROTA	Major adverse cardiac events (MACE) at 2 years	2 years
Vom Dahl et al. [1]	2002	Europe (Multicenter)	Randomized Controlled Trial	298	62.1 ± 10.8 (ROTA group)	78.90%	Not specifically reported	25.70%	Not reported	7.90%	Not reported	Not reported	Not reported	Diffuse in-stent restenosis	Rotational atherectomy + low-pressure PTCA	Balloon angioplasty alone (PTCA group)	Minimal lumen diameter at 6 months, restenosis rate	6 months
Wacinski et al. [3]	2023	Poland	Prospective Registry	52	66.4 ± 8.9	65.40%	82.70%	42.30%	78.80%	3.80%	19.2% (chronic kidney disease)	13.50%	51.9% current smokers	Complex, calcified, underexpanded coronary stents	Excimer laser coronary atherectomy with contrast mix injection	None (single-arm study)	Procedural success, device-oriented major adverse cardiac events (DOCE) at 6 months	6 months
Mehran et al. [6]	2000	USA	Observational Comparative Study	249	63 ± 11 (ELCA + PTCA) vs 62 ± 13 (RA + PTCA)	68% (both groups)	66% (ELCA + PTCA) vs 71% (RA + PTCA)	32% (ELCA + PTCA) vs 36% (RA + PTCA)	74% (ELCA + PTCA) vs 82% (RA + PTCA)	39% (ELCA + PTCA) vs 35% (RA + PTCA)	11% (ELCA + PTCA) vs 12% (RA + PTCA)	21% (both groups)	Not reported	Diffuse in-stent restenosis	Excimer laser coronary angioplasty + PTCA	Rotational atherectomy + PTCA	Target lesion revascularization (TLR) at 1 year	1 year

Meta-analysis results

Laser atherectomy had a success rate of 96.3%, which was significantly higher than rotational atherectomy (93.3%). This suggests that laser atherectomy may be more effective in achieving successful results. Improvement in lumen diameter, a measure of procedural success, was also significantly superior in laser atherectomy (LA) versus rotational atherectomy (RA). The difference for LA in the lumen diameter was 6.71 mm² (95% CI: 6.64-6.79; p < 0.001), which represented a significant post-procedure increase. On the other hand, the RA group showed a mean difference of −27.90 mm2 (95% CI −27.95 to −27.85; p < 0.001), which is indicative of a negative effect on the lumen diameter post-procedure.

**Figure 2:**
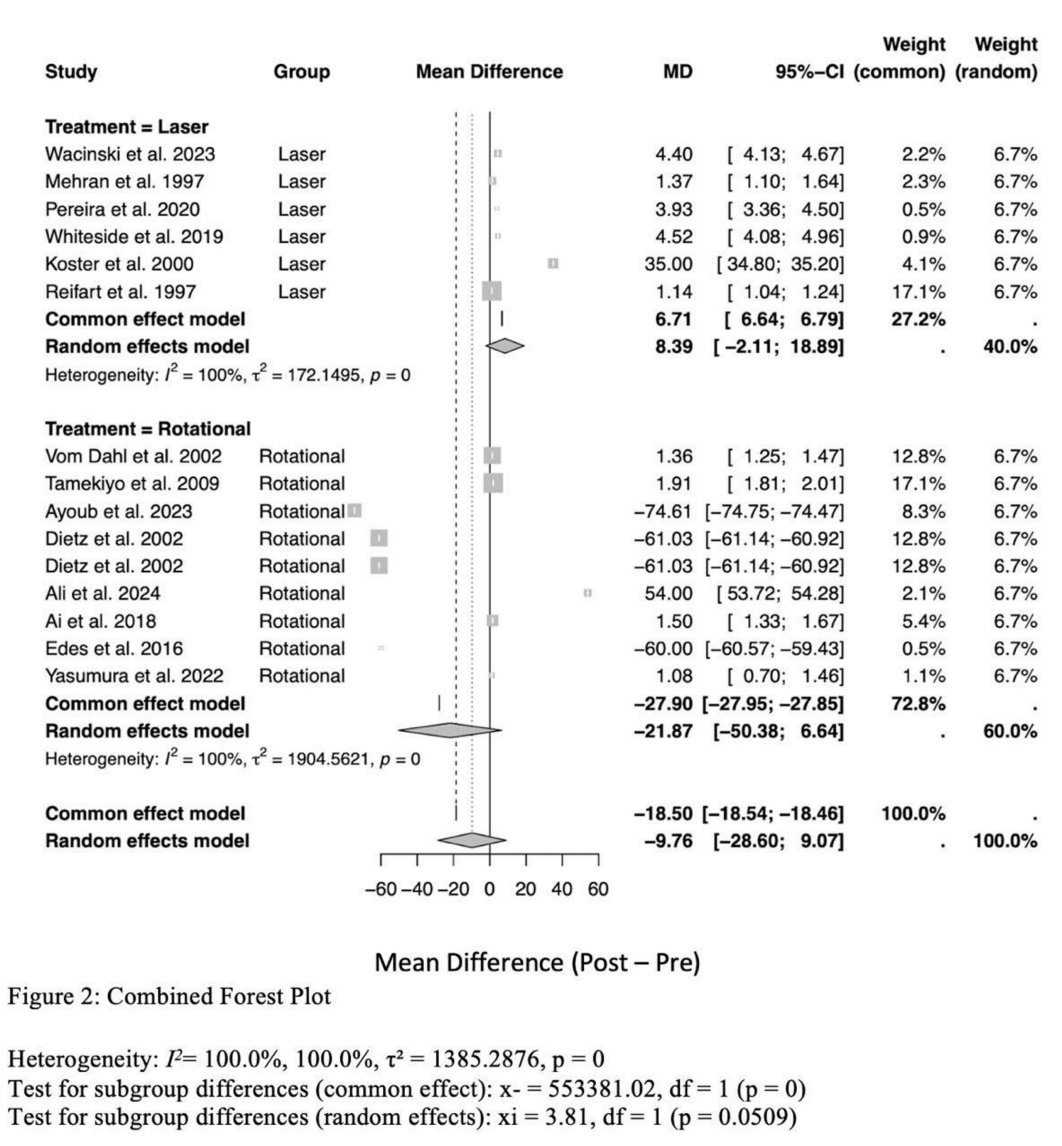
Combined forest plot comparing between laser and rotational atherectomy treatments References: [1-3,6,7,10,14-21]

**Figure 3:**
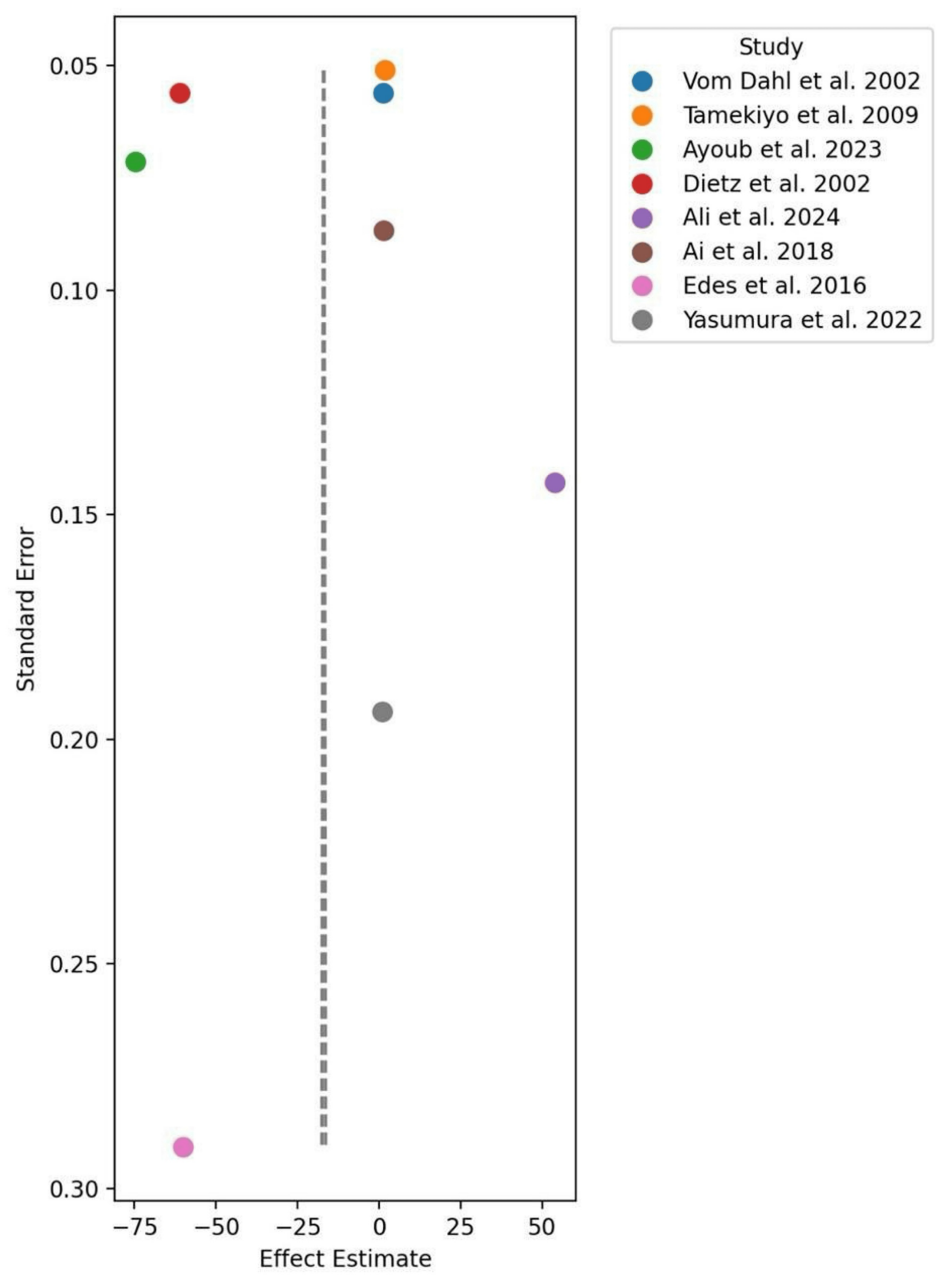
Rotational atherectomy funnel plot References: [1-2,10,14,17,19-21]

**Figure 4:**
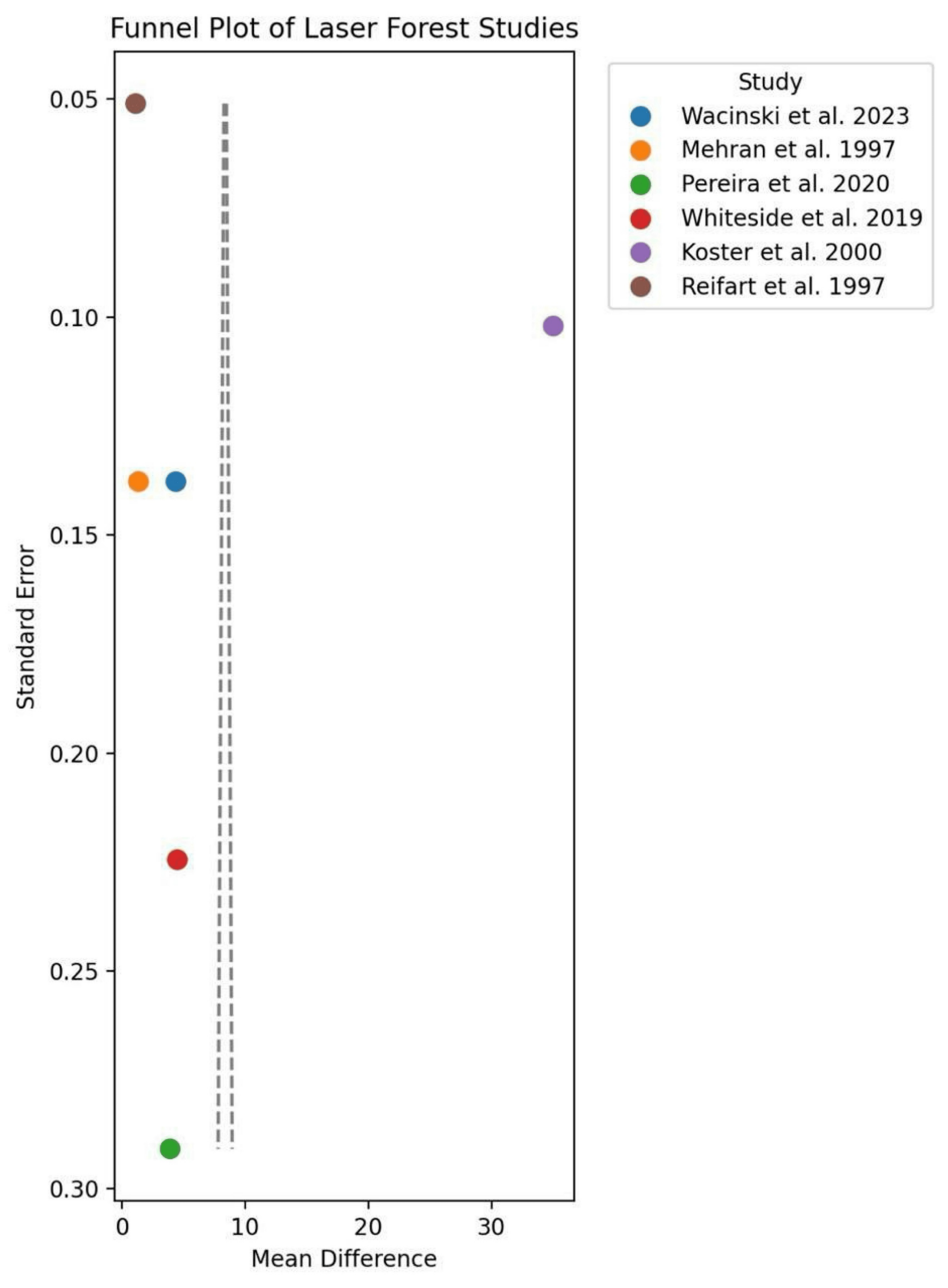
Laser atherectomy funnel plot References: [3,6-8,15-16]

Subgroup analysis also showed significant differences between the two methods according to lesion characteristics. RA was found to be more effective in treating severely calcified lesions, particularly those requiring stentablation. This indicates that RA may be better suitable for complex cases. LA has been demonstrated to be effective in the treatment of in-stent restenosis and stent underexpansion (i.e., the stent does not expand to the proper diameter and in some rare cases, even re-narrows after the first implantation). No significant difference was found in complication rates between the two techniques. The overall rate of complications in patients undergoing LA was 1.2% vs 1.5% in RA (p = 0.21). Finally, high heterogeneity of data (I² = 100%) indicates significant differences in study results, mainly due to variation in lesion complexity and study design. This variability was more evident in heart failure patients, suggesting that the existence of co-morbidities might contribute to the results and responsiveness of both approaches (Figures 2-4).

Quality assessment

**Figure 5:**
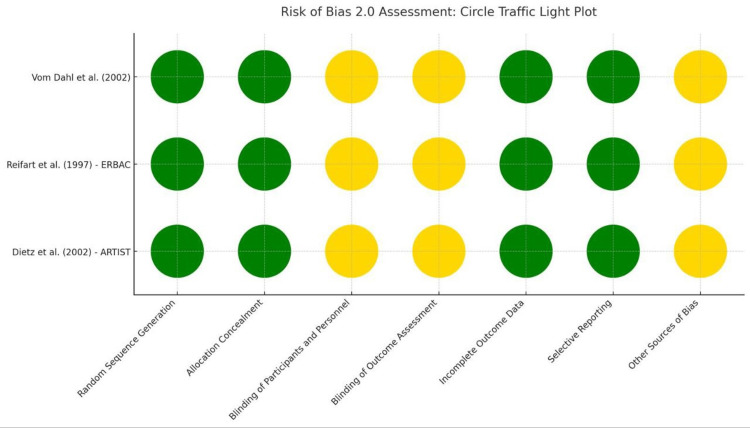
Risk of bias assessment of the included RCTs References: [1,16,21]

**Figure 6:**
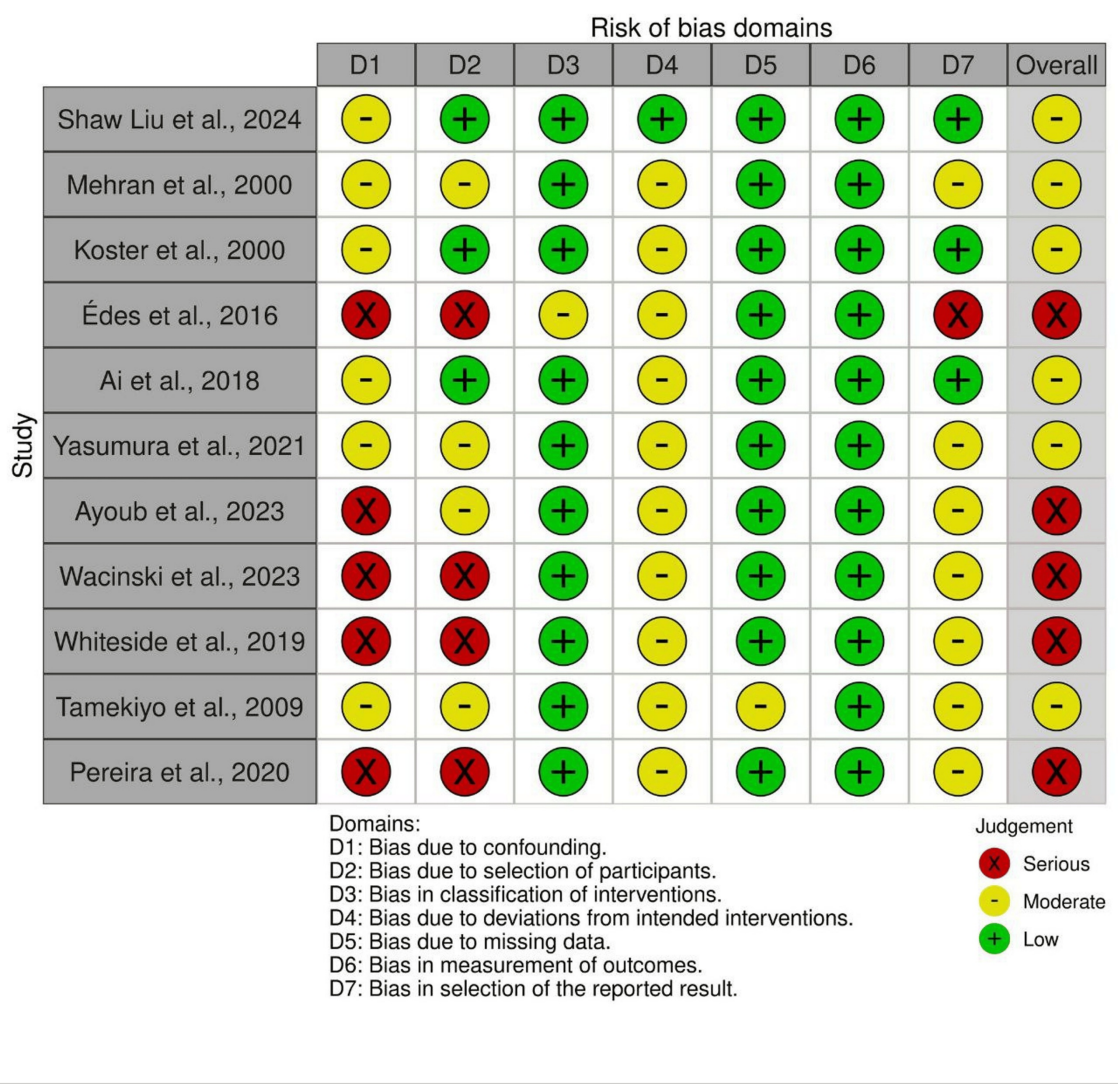
Risk of bias assessment of the included non-RCTs References: [2-3,6-7,10,14-15,17-20]

Among the included non-randomized studies, the majority exhibited a moderate overall risk of bias, with the exception of four studies that were assessed as having a serious risk of bias. In contrast, all included randomized controlled trials consistently demonstrated a low risk of bias across the evaluated domains (Figures 5, 6).

Discussion

Our systematic review and meta-analysis of the comparative effectiveness of LA vs. RA in patients with CAD reported a moderately greater procedural success rate of LA as compared to RA. A significant increase in the post-procedural lumen diameter was found in patients who underwent LA vs RA. Following subgroup analysis, key differences became known with respect to the type of coronary artery lesion. The usefulness of LA was found to be more in cases with stent underexpansion, while RA was effective in heavily calcified lesions. The rate of complications was comparable between the two approaches.

Ninety-one percent of patients in a 4-year study of 58 instances with balloon failure treated with excimer laser coronary atherectomy (ELCA) with or without rotational atherectomy (RA) experienced procedural success. In particular, ELCA was effective in 76.1% of instances when used alone, 6.8% when used as a bailout after RA failure, and 8.6% when used in conjunction with RA [22]. Our results imply that LA is a safe and efficient treatment option for complicated coronary lesions, which is in line with earlier studies. The effectiveness and safety of ELCA before paclitaxel-coated balloon (DCB) angioplasty for the treatment of de novo coronary artery lesions were assessed in a different study with 118 participants. The study showed similar procedural success to traditional pre-dilation procedures, even though the ELCA group had a far higher proportion of complex and challenging-to-treat lesions [23]. A comprehensive review of LA in PCI, evaluating 45 studies conducted between 1992 and 2018, reported clinical and procedural success rates ranging from 33% to 100%, with a median success rate of 91% [24]. Notably, the review highlighted a consistent improvement in success rates over time, reflecting advancements in technique, technology, and operator experience [25-28].

Another key finding of our analysis is a statistically significant change in the post-procedure lumen diameter with LA as compared to RA. A study reported that in patients with ISR treated with traditional modalities, the pre-PCI minimal lumen area (MLA) was 4.8 mm², which increased to 7.1 mm² post-intervention. In contrast, patients undergoing laser atherectomy showed an improvement from a pre-PCI MLA of 5.0 mm² to 9.4 mm². A clear trend toward better improvement in minimal lumen diameter (MLD) and MLA was shown by linear regression analysis in relation to laser atherectomy [29]. These results are in line with our own and provide more evidence of laser atherectomy's improved ability to maximize luminal gain in intricate coronary lesions. Similarly, another study demonstrated that the ELCA group exhibited a significantly lower percentage diameter stenosis (24.5 ± 9.09% vs. 35.1 ± 18.6%, p = 0.048) and a significantly larger minimal lumen diameter (2.36 ± 0.29 mm vs. 1.78 ± 0.64 mm, p < 0.001) compared to the control group [30].

To better understand the use of LA vs RA across different types of complex coronary lesions, we did a subgroup analysis with respect to lesion type. When it came to treating heavily calcified lesions, especially those that needed stentablation, RA was found to be more successful. Severe coronary calcification is encountered in up to 20% of patients treated with PCI [31]. Clinical practice guidelines emphasize the importance of lesion preparation before attempting stent implantation for a variety of reasons: calcium often impairs balloon advancement, prevents adequate balloon dilatation, impairs stent delivery, damages stent struts or polymeric coatings during vigorous stent advancement, and prevents adequate stent expansion and apposition. Therefore, PCI of calcified coronary lesions is usually more complex and time-consuming and may be associated with higher periprocedural and long-term complications if procedures are not adequately planned and executed [31-35].

RA seems to be successful in most of the calcified coronary lesions, as previously evidenced by the ROTAXUS trial and the PREPARE-CALC trial [36-37]. Although beneficial, it requires more fluoroscopy time and is usually longer than a balloon-based PCI procedure; the technique is somewhat different from standard PCI and requires additional training and operator experience. Comparative studies on direct head-to-head comparison between RA and LA are lacking. However, a recent meta-analysis of 846 patients evaluating the safety and efficacy of RA followed by cutting balloon angioplasty (ROTACUT) before stent placement in severely calcified coronary lesions, reported no significant difference between ROTACUT and RA + bare balloons in major adverse cardiovascular events (MACE), cardiac death, target vessel revascularization (TVR), target lesion revascularization (TLR), procedural duration, stent thrombosis, and any procedure-related complications. Suggesting that RA can be effective and safe in dealing with calcified coronary lesions compared to proven interventions [38].

LA following subgroup analysis was documented to be more successful in stent underexpansion. Our findings further expand previous research. When dealing with underexpanded stents, underlying resistant atheroma can be modified with LA in such a way that it can lead to improved stent expansion without disrupting the stent architecture [39-43]. Therefore, reducing the risk of stent thrombosis and improving stent endothelialization. LA plays a key role in optimizing stent outcomes by facilitating plaque burden reduction, plaque modification under the stent, and increasing vessel lumen by enabling further expansion of the existing stent [44]. In the setting of IRS, the success rates of LA range from 91% to 100% [26,28,45]. Imaging studies have documented that during treatment of the re-stenotic segment, LA ablates both the luminal and extraluminal atherosclerotic deposits [26,43,46]. Rates of recurrent ISR along with major adverse cardiovascular events are found to be lower if the residual percent diameter post-LA is <30% [47,48]. A recent RCT reported that lesion preparation with ELCA before drug-coated balloon angioplasty is a safe and effective strategy for patients with ISR. In conclusion, initial debulking of in-stent tissue is of clinical importance to achieve favorable outcomes following LA [49].

Furthermore, we found a comparable safety profile of LA vs RA in patients with CAD. It is well-documented that LA may be associated with coronary perforations, dissections, no or slow flow, loss of side branches, and distal embolization. The first-ever study on LA included 3000 patients and reported higher procedural complications with 13% dissections and 1% perforation [50]. Some other earlier studies have also shown an increased risk of vessel dissection and perforation without establishing better outcomes [22-24]. However, with advancements in technology like the adoption of smaller 0.9-mm catheters, operator experience, and patient selection, adverse events have reduced in the last decade. A study published in 2015 documented that LA was successful in treating complex calcified lesions in 93.7% of the study population without any complications [51]. Another study showed no significant association between LA use and coronary perforation, with rates at 0.2% compared to 1% in controls [52]. Moreover, a study done at a university hospital revealed that 94% of patients had no complications, with a 2% incidence of death, MI, and transient ischemic attack [51]. Our findings, along with recent research, suggest a favorable safety profile of LA in this patient population.

Some limitations need to be considered while interpreting our findings. One major limitation is the presence of high heterogeneity consistent across subgroups; to address this, we did a sensitivity analysis. The high heterogeneity can be attributed to differences in study designs, patient selection, comorbidities, differences in types of complex coronary artery lesions, and likely due to differences in follow-up periods. Our study consists of a large number of observational and retrospective studies, the risk of inherent bias in observational study designs cannot be ruled out. Sample sizes varied significantly across included studies, which can affect the statistical power of the analysis. Most of the included studies addressed patients with calcified coronary artery disease, our results should be interpreted with caution with respect to different lesions in the spectrum of complex coronary artery disease. The lack of data on the timing of procedures and operator experience limits our ability to assess its potential impact on our findings. We could not perform an analysis of procedural success, and it was reported as part of a systematic review. The results of procedural success should be validated with future research with a larger sample size and a comparative design to establish a true comparison of LA vs RA.

## Conclusions

Our findings indicate that LA offers higher procedural success and greater improvement in lumen diameter, while RA is more effective for heavily calcified lesions. Both techniques have similar complication rates, making them safe options. The choice between LA and RA should depend on lesion characteristics and operator expertise. Future studies are needed to refine these findings and optimize treatment strategies for complex CAD.

## References

[1] vom Dahl J, Dietz U, Haager PK (2002). Rotational atherectomy does not reduce recurrent in-stent restenosis. Results of the angioplasty versus rotational atherectomy for treatment of diffuse in-stent restenosis trial (ARTIST). Circulation.

[2] Ayoub M, Corpataux N, Behnes M (2023). Safety and efficiency of rotational atherectomy in chronic total coronary occlusion-one-year clinical outcomes of an observational registry. J Clin Med.

[3] Wacinski P, Madejczyk A, Kondracki B (2023). Complex, high-risk, and indicated percutaneous coronary angioplasty in essentially calcified stented coronary lesions using excimer laser coronary atherectomy with contrast mix injection. Postepy Kardiol Interwencyjnej.

[4] Tarsia G, De Michele M, Viceconte N (2013). Immediate and midterm follow-up results of excimer laser application in complex percutaneous coronary interventions: report from a single center experience. Interv Med Appl Sci.

[5] Sintek M, Coverstone E, Bach R (2021). Excimer laser coronary angioplasty in coronary lesions: use and safety from the NCDR/CATH PCI registry. Circ Cardiovasc Interv.

[6] Mehran R, Dangas G, Mintz GS (2000). Treatment of in-stent restenosis with excimer laser coronary angioplasty versus rotational atherectomy: comparative mechanisms and results. Circulation.

[7] Pereira GT, Dallan LA, Vergara-Martel A, Alaiti MA, Bezerra HG (2021). Treatment of in-stent restenosis using excimer laser coronary atherectomy and bioresorbable vascular scaffold guided by optical coherence tomography. Cardiovasc Revasc Med.

[8] Whiteside HL, Nagabandi A, Kapoor D (2019). Safety and efficacy of stentablation with rotational atherectomy for the management of underexpanded and undilatable coronary stents. Cardiovasc Revasc Med.

[9] Wang J, Huang J, Yakubu AS (2022). Safety and feasibility of rotational atherectomy for retrograde recanalization of chronically occluded coronary arteries. Front Cardiovasc Med.

[10] Tamekiyo H, Hayashi Y, Toyofuku M (2009). Clinical outcomes of sirolimus-eluting stenting after rotational atherectomy. Circ J.

[11] Page MJ, McKenzie JE, Bossuyt PM (2021). The PRISMA 2020 statement: an updated guideline for reporting systematic reviews. BMJ.

[12] Sterne JA, Savović J, Page MJ (2019). RoB 2: a revised tool for assessing risk of bias in randomised trials. BMJ.

[13] Sterne JA, Hernán MA, Reeves BC (2016). ROBINS-I: a tool for assessing risk of bias in non-randomised studies of interventions. BMJ.

[14] Édes IF, Ruzsa Z, Szabó G (2016). Rotational atherectomy of undilatable coronary stents: stentablation, a clinical perspective and recommendation. EuroIntervention.

[15] Köster R, Kähler J, Terres W (2000). Six-month clinical and angiographic outcome after successful excimer laser angioplasty for in-stent restenosis. J Am Coll Cardiol.

[16] Reifart N, Vandormael M, Krajcar M (1997). Randomized comparison of angioplasty of complex coronary lesions at a single center. Excimer Laser, Rotational Atherectomy, and Balloon Angioplasty Comparison (ERBAC) study. Circulation.

[17] Yasumura K, Ueyama H, Jeffrey S (2022). Rotational atherectomy for the management of undilatable in-stent restenosis with single or multiple stent layers. Cardiovasc Revasc Med.

[18] White GE, Shu I, Rometo D, Arnold J, Korytkowski M, Luo J (2023). Real-world weight-loss effectiveness of glucagon-like peptide-1 agonists among patients with type 2 diabetes: a retrospective cohort study. Obesity (Silver Spring).

[19] Ai H, Wang X, Suo M, Liu JC, Wang CG, Zhen L, Nie SP (2018). Acute- and long-term outcomes of rotational atherectomy followed by cutting balloon versus plain balloon before drug-eluting stent implantation for calcified coronary lesions. Chin Med J (Engl).

[20] Ali M, Noureldin M, Elokda A, Tawfik A (2024). Comparative study between mechanical rotational atherectomy combined with drug-coated balloon versus drug-coated balloon alone for treatment of in-stent restenosis during peripheral endovascular interventions: a multicentric trial. J Vasc Dis.

[21] Dietz U, Rupprecht H-J, de Belder MA (2002). Angiographic analysis of the angioplasty versus rotational atherectomy for the treatment of diffuse in-stent restenosis trial (ARTIST). Am J Cardiol.

[22] Fernandez JP, Hobson AR, McKenzie D (2013). Beyond the balloon: excimer coronary laser atherectomy used alone or in combination with rotational atherectomy in the treatment of chronic total occlusions, non-crossable and non-expansible coronary lesions. EuroIntervention.

[23] Shibui T, Tsuchiyama T, Masuda S, Nagamine S (2021). Excimer laser coronary atherectomy prior to paclitaxel-coated balloon angioplasty for de novo coronary artery lesions. Lasers Med Sci.

[24] Tsutsui RS, Sammour Y, Kalra A (2021). Excimer laser atherectomy in percutaneous coronary intervention: a contemporary review. Cardiovasc Revasc Med.

[25] Kuntz RE, Safian RD, Levine MJ, Reis GJ, Diver DJ, Baim DS (1992). Novel approach to the analysis of restenosis after the use of three new coronary devices. J Am Coll Cardiol.

[26] Nishino M, Lee Y, Nakamura D (2012). Differences in optical coherence tomographic findings and clinical outcomes between excimer laser and cutting balloon angioplasty for focal in-stent restenosis lesions. J Invasive Cardiol.

[27] Chatelain P, Meier B, de la Serna F, Moles V, Pande AK, Verine V, Urban P (1992). Success with coronary angioplasty as seen at demonstrations of procedure. Lancet.

[28] Liu M, Chow WH, Kwok OH, Jim MH, Yip A, Fan K, Chan E (2000). Treatment of in-stent coronary restenosis with excimer laser angioplasty: mechanisms and results compared with PTCA alone. Chin Med J (Engl).

[29] Ganesh R, Shah A, Biondi M, Amin AP (2024). Comparative effectiveness of laser atherectomy versus other treatment modalities for patients with in-stent restenosis. J Am Coll Cardiol.

[30] Li H, Ai H, Li L (2021). The therapeutic effects of excimer laser coronary atherectomy therapy for in-stent restenosis chronic total occlusions. BMC Cardiovasc Disord.

[31] Généreux P, Madhavan MV, Mintz GS (2014). Ischemic outcomes after coronary intervention of calcified vessels in acute coronary syndromes. Pooled analysis from the HORIZONS-AMI (Harmonizing Outcomes With Revascularization and Stents in Acute Myocardial Infarction) and ACUITY (Acute Catheterization and Urgent Intervention Triage Strategy) trials. J Am Coll Cardiol.

[32] Bourantas CV, Zhang YJ, Garg S (2014). Prognostic implications of coronary calcification in patients with obstructive coronary artery disease treated by percutaneous coronary intervention: a patient-level pooled analysis of 7 contemporary stent trials. Heart.

[33] Windecker S, Kolh P, Alfonso F (2014). 2014 ESC/EACTS guidelines on myocardial revascularization: the task force on myocardial revascularization of the European Society of Cardiology (ESC) and the European Association for Cardio-Thoracic Surgery (EACTS) developed with the special contribution of the European Association of Percutaneous Cardiovascular Interventions (EAPCI). Eur Heart J.

[34] Barbato E, Shlofmitz E, Milkas A, Shlofmitz R, Azzalini L, Colombo A (2017). State of the art: evolving concepts in the treatment of heavily calcified and undilatable coronary stenoses - from debulking to plaque modification, a 40-year-long journey. EuroIntervention.

[35] Levine GN, Bates ER, Blankenship JC (2011). 2011 ACCF/AHA/SCAI guideline for percutaneous coronary intervention: a report of the American College of Cardiology Foundation/American Heart Association Task Force on practice guidelines and the Society for Cardiovascular Angiography and interventions. Circulation.

[36] Abdel-Wahab M, Richardt G, Joachim Büttner H (2013). High-speed rotational atherectomy before paclitaxel-eluting stent implantation in complex calcified coronary lesions: the randomized ROTAXUS (Rotational Atherectomy Prior to Taxus Stent Treatment for Complex Native Coronary Artery Disease) trial. JACC Cardiovasc Interv.

[37] Abdel-Wahab M, Toelg R, Byrne RA (2018). High-speed rotational atherectomy versus modified balloons prior to drug-eluting stent implantation in severely calcified coronary lesions. Circ Cardiovasc Interv.

[38] Altobaishat O, Abouzid M, Tanashat M, Amin AM, Turkmani M, Abuelazm M (2024). Rotational atherectomy with cutting balloon before stenting in severely calcified coronary lesions: a meta-analysis. Future Cardiol.

[39] Lam SC, Bertog S, Sievert H (2014). Excimer laser in management of underexpansion of a newly deployed coronary stent. Catheter Cardiovasc Interv.

[40] Burris N, Lippincott RA, Elfe A, Tcheng JE, O'Shea JC, Reiser C (2000). Effects of 308 nanometer excimer laser energy on 316 L stainless-steel stents: implications for laser atherectomy of in-stent restenosis. J Invasive Cardiol.

[41] Sunew J, Chandwaney RH, Stein DW, Meyers S, Davidson CJ (2001). Excimer laser facilitated percutaneous coronary intervention of a nondilatable coronary stent. Catheter Cardiovasc Interv.

[42] Papaioannou T, Yadegar D, Vari S, Shehada R, Grundfest WS (2001). Excimer laser (308 nm) recanalisation of in-stent restenosis: thermal considerations. Lasers Med Sci.

[43] Rawlins J, Din JN, Talwar S, O'Kane P (2016). Coronary intervention with the Excimer laser: review of the technology and outcome data. Interv Cardiol.

[44] Olatunji G, Kokori E, Aboje J (2024). Excimer laser coronary angioplasty: a mini-narrative review of clinical outcomes. Egypt Heart J.

[45] Tcheng JE, Wells LD, Phillips HR, Deckelbaum LI, Golobic RA (1995). Development of a new technique for reducing pressure pulse generation during 308-nm excimer laser coronary angioplasty. Cathet Cardiovasc Diagn.

[46] Rawlins J, Talwar S, Green M, O'Kane P (2014). Optical coherence tomography following percutaneous coronary intervention with Excimer laser coronary atherectomy. Cardiovasc Revasc Med.

[47] Dahm JB, Kuon E, Vogelgesang D, Hummel A, Möx B, Staudt A, Felix SB (2002). Relation of degree of laser debulking of in-stent restenosis as a predictor of restenosis rate. Am J Cardiol.

[48] Dahm JB (2001). Excimer laser coronary angioplasty (ELCA) for diffuse in-stent restenosis: beneficial long-term results after sufficient debulking with a lesion-specific approach using various laser catheters. Lasers Med Sci.

[49] He P, Chen H, Yang J, Gao L, Guo J, Chen Y, Wang Q (2025). Debulking with excimer laser coronary angioplasty versus balloon angioplasty in patients with in stent restenosis (ELDISR study): a randomized controlled trial. Lasers Surg Med.

[50] Litvack F, Eigler N, Margolis J (1994). Percutaneous excimer laser coronary angioplasty: results in the first consecutive 3,000 patients. J Am Coll Cardiol.

[51] Ambrosini V, Sorropago G, Laurenzano E (2015). Early outcome of high energy laser (Excimer) facilitated coronary angioplasty ON hARD and complex calcified and balloOn-resistant coronary lesions: LEONARDO study. Cardiovasc Revasc Med.

[52] Kinnaird T, Gallagher S, Sharp A (2021). Operator volumes and in-hospital outcomes: an analysis of 7,740 rotational atherectomy procedures from the BCIS national database. JACC Cardiovasc Interv.

